# Six Immune Associated Genes Construct Prognostic Model Evaluate Low-Grade Glioma

**DOI:** 10.3389/fimmu.2020.606164

**Published:** 2020-12-21

**Authors:** Yin Qiu Tan, Yun Tao Li, Teng Feng Yan, Yang Xu, Bao Hui Liu, Ji An Yang, Xue Yang, Qian Xue Chen, Hong Bo Zhang

**Affiliations:** ^1^ Department of Neurosurgery, Renmin Hospital of Wuhan University, Wuhan, China; ^2^ Department of Neurosurgery, Zhujiang Hospital, Southern Medical University, Guangzhou, China; ^3^ The National Key Clinical Specialty, The Engineering Technology Research Center of Education Ministry of China, Guangdong Provincial Key Laboratory on Brain Function Repair and Regeneration, Guangzhou, China

**Keywords:** glioma, tumor associated macrophage, single cell sequence, prognosis, biomarker

## Abstract

**Background:**

The immunotherapy of Glioma has always been a research hotspot. Although tumor associated microglia/macrophages (TAMs) proves to be important in glioma progression and drug resistance, our knowledge about how TAMs influence glioma remains unclear. The relationship between glioma and TAMs still needs further study.

**Methods:**

We collected the data of TAMs in glioma from NCBI Gene Expression Omnibus (GEO) that included 20 glioma samples and 15 control samples from four datasets. Six genes were screened from the Differential Expression Gene through Gene ontology (GO) analysis, Kyoto Encyclopedia of Genes and Genomes (KEGG) pathway analysis, protein–protein interaction (PPI) network and single-cell sequencing analysis. A risk score was then constructed based on the six genes and patients’ overall survival rates of 669 patients from The Cancer Genome Atlas (TCGA). The efficacy of the risk score in prognosis and prediction was verified in Chinese Glioma Genome Atlas (CGGA).

**Results:**

Six genes, including CD163, FPR3, LPAR5, P2ry12, PLAUR, SIGLEC1, that participate in signal transduction and plasma membrane were selected. Half of them, like CD163, FPR3, SIGLEC1, were mainly expression in M2 macrophages. FPR3 and SIGLEC1 were high expression genes in glioma associated with grades and IDH status. The overall survival rates of the high risk score group was significantly lower than that of the low risk score group, especially in LGG.

**Conclusion:**

Joint usage of the 6 candidate genes may be an effective method to diagnose and evaluate the prognosis of glioma, especially in Low-grade glioma (LGG).

## Introduction

Glioma is the most common primary tumor in central nervous system, accounting for 80% of all malignant brain tumors (1). Current glioma treatment frequently involves many ways, including surgery, radiation therapy, chemotherapy, immunotherapy (2), targeted therapy (3), and tumor treating fields (TTF) (4). Although modern aggressive comprehensive treatments are improving, the outcome for glioma remains quite poor. Gliomas are complexly composed of diverse malignant cells and nonmalignant cells, whose development in a special environment called tumor microenvironment (TME) (5). Among the myriad cell types, microglia, and infiltrating macrophages are known as tumor associated microglia/macrophages (TAMs), accounting for 30%~50% of the glioma mass (6). Through interactions with neoplastic cells, TAMs provide a tumor-favorable microenvironment that enable glioma to escape immune surveillance, consequently promoting glioma proliferation and metastasis (6). Therefore it is important to improve our understanding of the interactions between glioma and TAMs and then to develop more effective treatment strategies.

The TAMs of glioma are composed of two distinct populations, including tissue-resident microglia and bone marrow-derived macrophages (BMDMs) (7). According to the cell markers and functions of TAMs, they are divided into two phenotypes: the M1 macrophages phenotype is associated with inflammation playing a role in anti-tumor, while the M2 macrophages phenotype mediate the tumor growth by promoting the secretion of angiogenesis factor and immunosuppressive cytokine (8). *In vitro*, the similar dual phenotypes have been induced by exposure either to LPS/IFNγ or IL10/IL4 (9). More recently, the complex situation of TAMs had been discussed extensively and discovered the current M1 and M2 classification schemes are not absolute, other classifications based on the specific pathways or molecules are used to identify the phenotypes of TAMs (10). Whereas many research have revealed that the strategies converting M2 macrophages to M1 macrophages or inhibiting M2-polarization of TAMs suppressed tumor growth (11). However, the communication between glioma and TAMs is still unclear. To understand the glioma comprehensively and deeply, the study of TAMs is essential.

Here, we screened bulk-RNA sequencing and Single-cell-RNA sequencing data that compare TAMs of glioma with normal microglia collected from non-tumor samples from GEO database, analyzed the differential expression genes (DEGs) and then tested the relationship between DEGs and prognosis of glioma by using data from TCGA and CCGA. We found most of the DEGs between TAMs and non-tumor microglia are also the different genes between M1 and M2 macrophages. However the prognosis of low-grade glioma cannot be predicted by single gene from the DEGs passed through screening.Finally, we constructed a risk score based on the six genes by using TCGA database and verified it in CGGA database. Meanwhile we explored the role of SIGLEC1 (also known as CD169) and FPR3 in the prognosis and immunotherapy of glioma and thought them would be new biomarkers and targets in diagnoses and treatment of glioma.

## Materials and Methods

### Patient Samples

The Ethics Committee of Wuhan University approved this study, and all experiments complied with the current laws of PR China. In total, three control samples from patients with cerebral hemorrhage and six glioma samples were collected during May 2020 and October 2020, including both low-grade glioma (grade I, one case; grade II, two cases) and glioblastoma multiform (grade IV, three cases). All patients provided written informed consent. Samples of tumor tissue were collected during surgery, snap-frozen in liquid nitrogen, and stored until experimental use. Patients were not treated with chemotherapy or radiotherapy before surgery.

### Data Acquisition

This study acquired 20 glioma samples and 15 control samples from four datasets downloaded from NCBI Gene Expression Omnibus(GEO)(https://www.ncbi.nlm.nih.gov/geo/), including GSE80338, GSE115397, GSE135437, and GSE84465. The gene expression data and clinical data including grades, IDH status and survival time are downloaded from TCGA (669 patients) (https://www.cancer.gov/) and CGGA (1018 patients) (http://www.cgga.org.cn/) database.

### Analysis of Differential Expression Gene

The bulk-RNASeq data was analyzed by limma package, while the scRNASeq data was analyzed by FindMarkers function of Seurat package. The DEGs in each of the three datasets were defined by using fold-change filtering (fold change >1) and padj <0.05, and then the up-regulated genes and down-regulated genes from each datasets were intersected, respectively.

### GO and KEGG Pathway Analysis

The functions of the 64 DEGs were uploaded to DAVID database (https://david.ncifcrf.gov/) to be analyzed. Hierarchical clustering of the DEGs was performed according to the biological process, cell component, molecular function and KEGG pathways. The terms were in rank according to the counts and p-value <0.05 was thought significance.

### Identification of Cell Types

Two scRNASeq data were pretreatment through the standard analysis process of Single cell analysis R package Seurat. Identification of cell types used specific cell markers acquired from the official CellMarker website (http://biocc.hrbmu.edu.cn/CellMarker/).

### ICH Images Acquisition

The ICH images of normal brain and glioma were acquired from THE HUMAN PROTEIN ATLAS (https://www.proteinatlas.org/), Due to the lack of protein expression data of FPR3 in brain and glioma, we acquired the proteins expression data of the rest five genes and the four levels are distinguished according to the degree of staining, including High, Medium, Low, and Not detected. The number of patients with staining also acquired.

### Quantitative Real-Time PCR and RNA Extraction

The extraction of total RNA from tissues and cells was carried out using the Trizol reagent (Invitrogen, USA). For the reverse transcription of RNA, the PrimeScript RT Reagent Kit (RR047A, Takara, Japan) was used to synthesize cDNA. Using SYBR Premix Ex Taq II (RR820A, Takara), we performed qPCR to detect mRNA levels following the specifications provided by the manufacturers. qPCR was performed on a 2.1 Real-Time PCR System using Bio-Rad CFX Manager (Bio-Rad, USA). The relative Ct method was adopted to compare the data of the experimental group and the control group, and β-actin was set as internal control. The primer sequences are listed in **Supplementary Table S2**.

### Statistical Analysis

mRNA expression, 2^-ΔΔCT^, as measured using RT-PCR in the different samples, was compared using One-way analysis of variance (ANOVA). Statistical analyses and visualization were performed in R 3.6.0. All the packages used in R were listed below: Cairo, ggplot2, ggplotify, Seurat, cowplot, survminer, survival, glmnet, ROCR, estimate, ggcorrplot, and ggpubr. The log-rank test was used in Kaplan-Meier survival analysis. Lasso regression was used to constructed prognostic model. Statistical significance was indicated in the figures as follows: ns *p* > 0.05, *p < 0.05, **p < 0.01, ***p < 0.001, ****p <= 0.0001.

## Results

### Sixty-Four Genes Were Associated With the TAMs of Glioma

**Figure 1:**
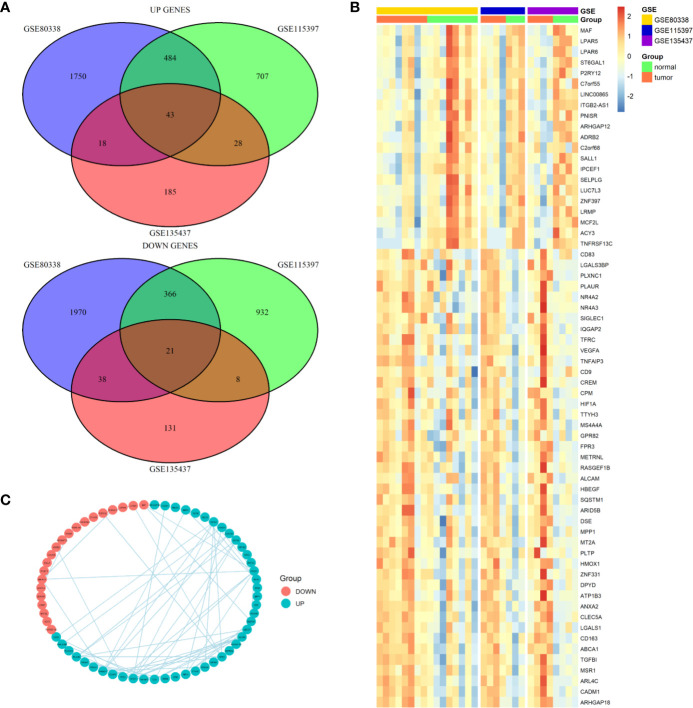
Differential expression genes profiles in microglia/macrophage from glioma and normal brain tissue. **(A)** The overlapping significantly differentially expressed genes in microglia/macrophage of glioma vs. normal. There were significantly 43 upregulated and 21 downregulated genes in microglia/macrophage of glioma vs. normal. **(B)** Hierarchical clustering of the differentially expressed genes in three datasets. **(C)** PPI network map showed the interaction of the 64 DEGs.

We first screened the GEO database and collected three datasets of TAMs in glioma, the GSE80338 and GSE115397 collected CD11b+ microglia/macrophages from glioma and normal brain tissue and sequenced using RNA sequencing, while the GSE135437 was using FACS sorted on lineage-negative live CD45-positive cells and sequenced using the mCEL-Seq2 protocol. The DEGs in each of the three datasets were defined by using fold-change filtering (fold change >1) and padj <0.05, the up-regulated genes and down-regulated genes from each datasets were intersected respectively. Finally, we got 43 up-regulated genes and 21 down-regulated genes (**Figure 1A**). A heatmap showed the expression of all this 64 DEGs in three datasets (**Figure 1B**). Among the 64 DEGs, we found many oncogenes such as HIF1A, VEGFA, TGFBI, and HBEGF. Meanwhile many immune cell markers were also included, like MAF, SALL1, MCF2L, CD83, CD163, and MSR1. A PPI network plot showed the interaction of the 64 DEGs (**Figure 1C**).

### GO and KEGG Pathway Analysis of the 64 DEGs and the Relationship With M1/M2 Macrophage

**Figure 2:**
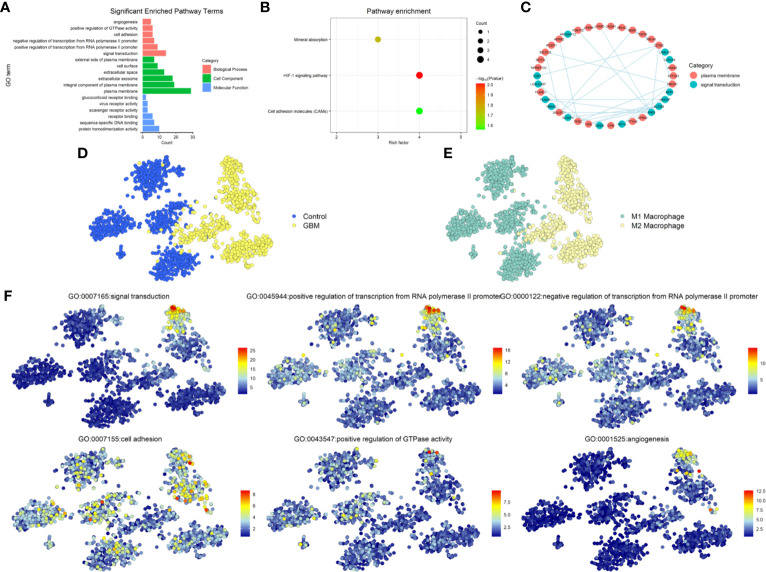
Functional enrichment analysis. **(A)** Gene ontology (GO) analysis for 64 DEGs. Pink indicates biological process (BP), green indicates molecular function (MF), and blue indicates cellular component (CC). **(B)** KEGG Pathway analysis for 64 DEGs and shows significantly enriched signaling pathways. **(C)** PPI network map showed the interaction of the 38 genes from cell adhesion, plasma membrane and Cell adhesion molecules. **(D)** The distribution of GBM and Control cells in GSE135437. **(E)** The distribution of status of macrophages in GSE135437. **(F)** The distribution of top-six biological processes in GSE135437.

To explore the function of the 64 DEGs, we performed GO and KEGG Pathway Analysis by uploading the DEGs into DAVID database. GO analysis showed the screened genes are involved in many important functions and structures. In BP category, most genes mainly enriched in signal transduction, rest of the DEGs enriched in the cell adhesion and angiogenesis that associated with the invasion and migration of glioma. In CC category, more than a third of the DEGs enriched in plasma membrane and integral component of plasma membrane. In the MF category, enriched terms included protein homodimerization activity, sequence-specific DNA binding, receptor binding, scavenger receptor activity, virus receptor activity, and glucocorticoid receptor binding (**Figure 2A**). The KEGG Pathway analysis revealed three pathways were involved such as Mineral absorption, HIF-1 signaling pathway and Cell adhesion molecules (CAMs) (**Figure 2B**). Due to the interaction between TAMs and glioma were mainly related to the signal transduction through the proteins in the plasma membrane and affect the invasion and migration of glioma, we narrowed the candidate genes down to 38 genes, subsequently choose the most interacted node genes, FPR3, and its interacted genes to explored further (**Figure 2C**).

According to the different biomarkers of M1/M2 macrophages, we defined the cell types of GSE135437 and studied the distribution of M1/M2 macrophages in GBM and control samples. Almost all M2 macrophages were in the GBM cells, while M1 macrophages were in the control cells (**Figures 2D, E** **)**. Furthermore we explored the relationship between the biological process and cell types and found that the signal transduction and angiogenesis enriched in a subgroup of M2 macrophages, however the cell adhesion widely distributed in both control and GBM cells (**Figure 2F**). To verify this relationship, we used another GBM scRNASeq dataset GSE84465 that including neoplastic cells, TAMs and many other types of cells in glioma. In contrast, the signal transduction mainly distributed in M1 macrophages, though part of M2 macrophages also expressed the signal transduction proteins. Meanwhile the cell adhesion signal was in the neoplastic cells and M2 macrophages. The angiogenesis signal was still in the M2 macrophages (**Supplementary Figure 1A**).

### The Distribution and Expression of Six Genes and the Relationship With M1/M2 Macrophage

**Figure 3:**
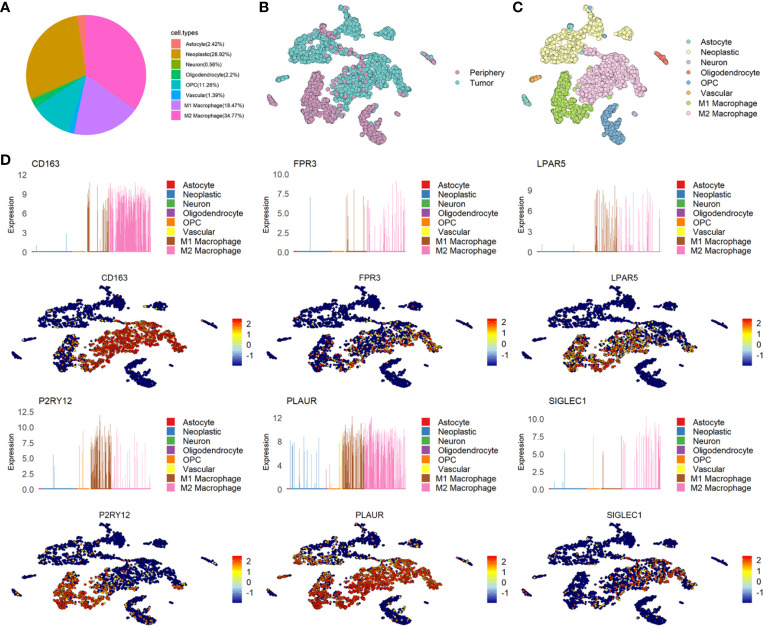
The distribution and expression of six candidate genes in glioma. **(A)** The percentage of each type of cells in glioma. **(B)** The distribution of Tumor and Periphery cells in GSE84465. **(C)** The distribution of each type of cells in glioma in GSE84465. **(D)** The distribution and expression of six candidate genes in GSE84465.

Microglia and macrophages take a major proportion of GBM. According to the cell annotation of GSE84465, nearly half of the cells were immune cells. We redefined the immune cells to subdivide into M1/M2 macrophages and found that 18.47% of the GBM cells were M1 macrophages, 34.77% of the GBM cells were M2 macrophages and 28.92% of the GBM cells were neoplastic cells (**Figure 3A**). Consistently with GSE135437, In GSE84465, almost all the M2 macrophages were in the GBM cells, while the M1 macrophages were in the periphery cells (**Figures 3B, C** **)**. Then we analyzed the distribution and expression of six genes, the results showed that CD163, FPR3, and SIGLEC1 were expressed almost exclusively in M2 macrophages, while LPAR5 was widely expressed in M1/M2 macrophages. In GSE84465, P2RY12 was mainly expression in M1 macrophages, but in another dataset, it expressed in both M1 and M2 macrophages. PLAUR was also not expressed in only one cell type (**Figure 3D** and **Supplementary Figure 1B**).

In consideration of the heterogeneity of GBM, each scRNASeq dataset only contained four couples of samples, we could not determine whether the difference between the two datasets reflected real features of the three genes. We determined to test the six genes in the TCGA and CCGA database.

### FPR3 and SIGLEC1, Two Novel Potential Diagnostic Biomarkers for Glioma

**Figure 4:**
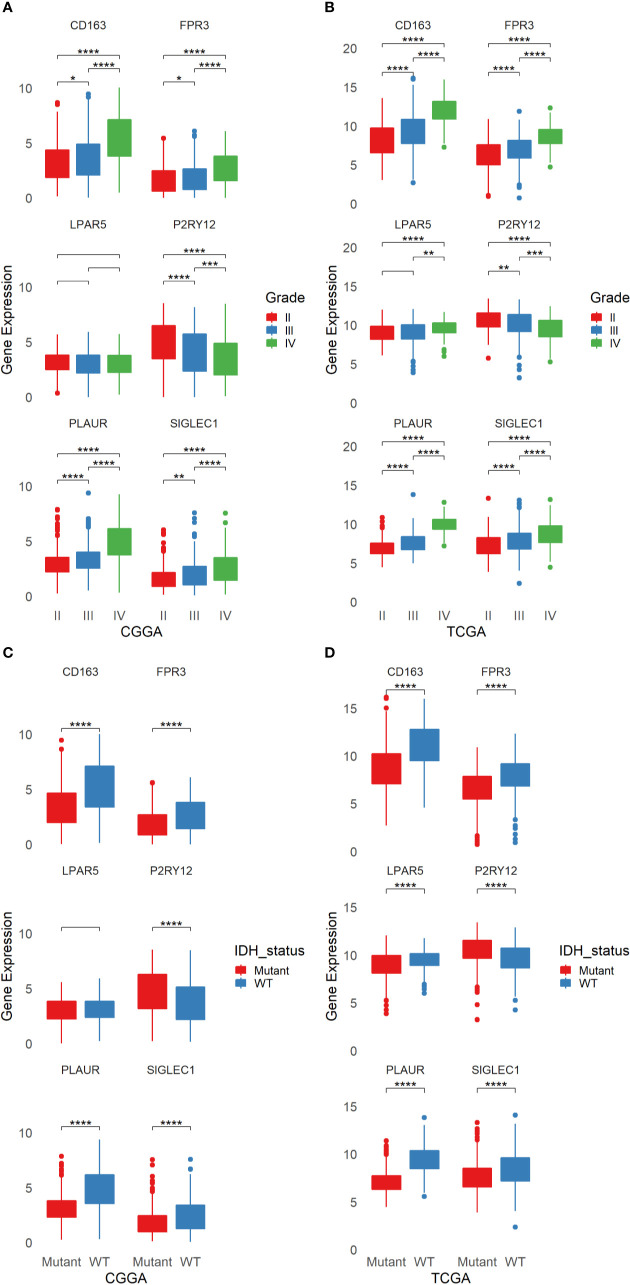
The Expression of six candidate genes in glioma from TCGA and CGGA. **(A)** The expression of six candidate genes in different grades of glioma from CGGA. **(B)** The expression of six candidate genes in different grades of glioma from TCGA. **(C)** The expression of six candidate genes in different status of IDH in glioma from CGGA. **(D)** The expression of six candidate genes in different status of IDH in glioma from TCGA. *p < 0.05, **p < 0.01, ***p < 0.001, ****p <= 0.0001.

The six genes were analyzed by using TCGA and CGGA database respectively. According to the tumor grades, IDH states, we tested all the six genes and found that the expression of LPAR5 had no differences in both tumor grades and IDH states in CGGA database, while in TCGA database, the expression of LPAR5 still had no differences between grade II and grade III. However, it can be used to differentiate glioma between grade II and grade IV. The differential expression of other five genes was significant and could be used to well distinguish among different grades and IDH states (**Figures 4A–D**).

Some researches had studied CD163, P2RY12 and PLAUR as biomarkers in glioma. Our TCGA and CGGA analysis results were consistent with the previous studies. However, the role of FPR3 and SIGLEC1 in glioma still not be explored. On account of the six genes were screened from immune cells, we divide gliomas into four groups in line with immune score and stromal score. Unfortunately, only the differential expression of CD163 in TCGA database can distinguish the high or low of immune score and stromal score, while FPR3 just only had a difference between the high score and low score of immune score in CGGA database (**Supplementary Figures 2A–D**).

The protein expression of six genes in glioma and normal brain were acquired from THE HUMAN PROTEIN ATLAS. However, no protein expression information of FPR3 in brain or glioma was found in the database. The expression of PLAUR and SIGLEC1 were not detected, while the expression of P2RY12 protein was high in both normal brain and glioma. The expression of CD163 and LPAR5 protein were lower in normal brain than glioma (**Supplementary Figure 4A**). The number of patients with staining of each protein was shown in **Supplementary Figure 4B**.

Then we performed qPCR to detect the mRNA expression of six genes in normal brain, LGG and GBM. The results revealed that the expression of CD163 and FPR3 were increasing in glioma, especially in GBM, the expression of P2RY12 was high in glioma, but more notable in LGG. SIGLEC1 was higher in GBM but not be detected in LGG. PLAUR was similar to SIGLEC1 and LPAR5 was higher in normal brain (**Supplementary Figure 4C**).

### Prognostic Model Based on Six Candidate Genes Well Evaluate the Prognosis of LGG

**Figure 5:**
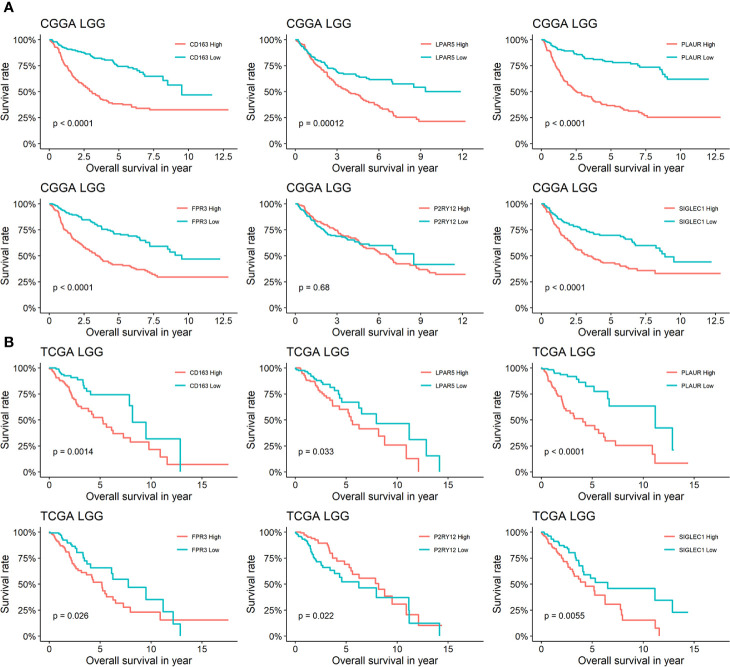
Survival analysis of six genes in LGG. **(A)** Kaplan-Meier curves for CD163, LPAR5, PLAUR, FPR3 P2RY12 and SIGLEC1 of LGG in CGGA. **(B)** Kaplan-Meier curves for CD163, LPAR5, PLAUR, FPR3 P2RY12, and SIGLEC1 of LGG in TCGA.

In order to analysis the effects of the six genes for prognosis in different grade glioma, We separated patients from TCGA and CGGA into four groups: TCGA LGG, TCGA GBM, CGGA LGG, and CGGA GBM. The analysis of TCGA LGG revealed that patients whose glioma expression high or low of LPAR5 had different outcomes, and consistent with LPAR5, the expression of other five genes all had a relationship with outcomes. The low expression of CD163, LPAR5, PLAUR, FPR3, and SIGLEC1 stand for a better outcomes and survival rates, while P2RY12 had the opposite outcomes (**Figure 5B**). Similarly, CD163, LPAR5, PLAUR, FPR3, and SIGLEC1 had the same effects in CGGA LGG, but P2RY12 had no effects (**Figure 5A**). However, only PLAUR could distinguish the prognosis of TCGA GBM (**Supplementary Figure 3B**) and CD163, PLAUR, and FPR3 could distinguish the prognosis of CGGA GBM (**Supplementary Figure 3A**).

**Figure 6:**
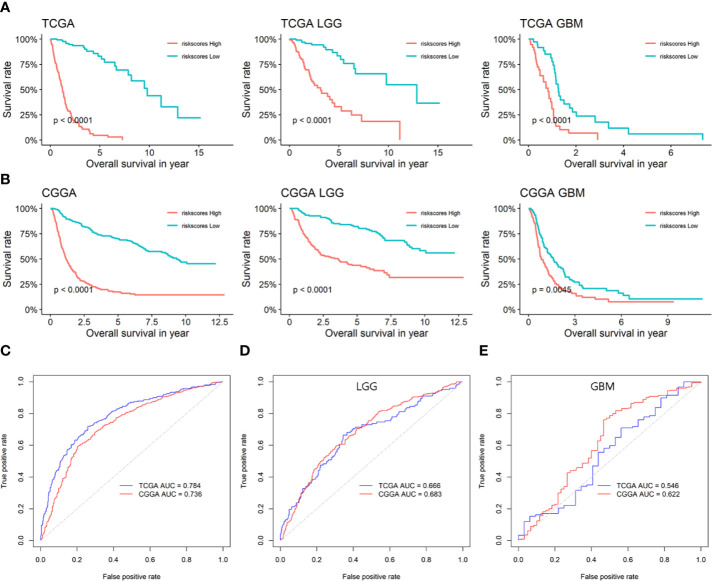
The prognostic efficiency of the six candidate genes and prognostic model. **(A)** Survival analysis of risk score in TCGA. **(B)** Survival analysis of risk score in CGGA. **(C)** ROC curves of the prognostic model based on the six candidate genes. **(D)** ROC curves of the prognostic model in LGG based on the six candidate genes. **(E)** ROC curves of the prognostic model in GBM based on the six candidate genes.

As glioma is a multi-gene disordered disease, we tried to construct a multi-gene model to evaluate the prognosis of LGG. Univariate/multivariate Cox regression analysis were performed to show the prognostic significance of six genes in LGG/GBM patients (**Table 1**). Lasso regression analysis was performed and risk score was calculated by the following formula: risk score = 0.15934970*expression(LPAR5)-0.03816307*expression(CD163)-0.07363766*expression(FPR3)-0.28186165*expression(P2RY12)+0.60211778*expression(PLAUR)+0.09642036*expression(SIGLEC1). The prognostic model was constructed by using TCGA data and verified in CGGA database. The AUC of TCGA and CCGA were 0.784 and 0.736, respectively (**Figure 6C**). K-M curves confirmed that the risk score could well predict the survival of both LGG and HGG patients (**Figures 6A, B** **)**. The AUC of LGG from TCGA and CCGA were 0.666 and 0.683, respectively (**Figure 6D**) and the AUC of GBM from TCGA and CCGA were 0.546 and 0.622, respectively (**Figure 6E**). The correlation between six genes and immune checkpoint also performed and shown in **Supplementary Figure 5**.

**Table 1:** Univariate/multivariate Cox regression analysis of six genes in LGG/GBM patients.

Gene	LGG					GBM				
	Univariate analysis			Multivariate analysis		Univariate analysis			Multivariate analysis	
	HR(95%CI)	P-value		HR(95%CI)	P-value	HR(95%CI)	P-value		HR(95%CI)	P-value
CD163	1.172(1.085-1.267)	<0.001*		0.934(0.832–1.049)	0.251	1.089(0.986–1.202)	0.093		0.903(0.765–1.065)	0.225
FPR3	1.166(1.051–1.293)	0.004*		0.889(0.773–1.023)	0.101	1.077(0.941–1.232)	0.281		0.971(0.772–1.220)	0.800
LPAR5	1.166(1.051–1.293)	0.004*		1.456(1.109–1.912)	0.007*	0.950(0.799–1.130)	0.561		0.851(0.572–1.267)	0.428
P2RY12	0.843(0.755–0.941)	0.002*		0.651(0.534–0.794)	<0.001*	0.893(0.794–1.004)	0.058		0.934(0.744–1.173)	0.558
PLAUR	0.843(0.755–0.941)	0.002*		1.576(1.313–1.893)	<0.001*	1.322(1.105–1.581)	0.002*		1.639(1.211–2.219)	0.001*
SIGLEC1	1.275(1.147–1.416)	<0.001*		1.161(1.015–1.329)	0.029*	1.060(0.949–1.183)	0.302		1.098(0.959–1.256)	0.177

CI, confidence interval; LGG, Low-grade glioma; GBM, Glioblastoma; HR, Hazard ratio.

*P < 0.05.

## Discussion

In recent years, many studied have highlighted the importance of tumor immune microenvironment in glioma and this has been the subject of intense research (12, 13). Despite the rapid development of tumor immunity research have promoted our understanding of glioma, the immunotherapy for glioma is still far from satisfactory (14). Thus, looking for more immune targets is still needed. Recently, many methods have emerged to predict glioma prognosis based on immune and stromal scores (15–18). Meanwhile, similar methods have been used in many other solid tumor studies to predict prognosis of patients (19–21). In previous studies, bulk RNASeq data downloaded from TCGA and CGGA were used to seek the immune-gene related signatures to evaluate the risk of LGG or GBM. We summarized some researches about immune-related gene to predict prognosis of LGG or GBM listed in **Table S1** **(** 15–18, 22–25). In consideration of the bias of bulk RNASeq data due to mixed cell type in tumor, we performed scRNASeq analysis to target TAMs and found 64 genes that differentially expressed between microglial and TAMs. Although many oncogenes are included in the DEGs, the interaction between TAMs and glioma thought to be taken place in plasma membrane, where cytokines and receptor combined and consequently changes the receptor cells to activate glioma and/or repress immune cell functions (26–28). So we narrowed the DEGs down to 38 genes that are contained in the signal transduction and plasma membrane. In addition, PPI network analysis showed that FPR3 had the most interacting proteins, such as CD163, P2RY12, LPAR5, PLAUR, and SIGLEC1. So we focused on this six genes and made a further research.

Previous studies have shown that CD163 is a biomarker that distinguish between M1 and M2 macrophages and correlated with survival times (29). Similar to our study, Liu (30) analysis a large scale glioma data and revealed that CD163 showed a positive relationship with immune cell populations in glioma and was up-regulated in IDH wild-type glioma. Meanwhile CD163 regulates the stemness of glioma (31), anti-PD-L1 antibody treatment significantly reduced infiltration of CD163+ macrophage in glioma (32). Hence, CD163 might serve as a therapeutic target for glioma. P2RY12 is also relevant to M1 and M2 macrophages according to its location in cells nuclear or cytoplasmic (33) and also differentially expressed between microglia and peripheral monocytes/macrophages in health and glioma (34). Otherwise, P2RY12 is involved in platelet aggregation (35) and is identified as key microglial surface marker (36). LPAR5 is one of the LPA receptor members, of which LPAR1 had been explored in glioma (37), but LPAR5 had been researched in promoting fibrosarcoma (38) and thyroid cancer (39). LPA signaling *via* LPA receptors contributes to the promotion of proliferation, invasion, and metastasis of tumor (40). Otherwise, LPA also regulate immune functions and inflammation (41). In papillary thyroid carcinoma, the LPAR5 is associated with immune infiltration (42). The function of LPAR5 in glioma still unclear, further research is still necessary. PLAUR encodes the urokinase receptor, which is influenced by hypoxia and promotes cell migration in GBM (43, 44). In polyautoimmunity, PLAUR contributes to regulation of apoptotic processes (45). The role of PLAUR is localizing and promoting plasmin formation (46), so the function of PLAUR may related to cell-surface plasminogen activation and localized degradation of the extracellular matrix. SIGLEC1 (also known as CD169), is also abnormal expression in peripheral macrophages of many cancers (47, 48), especially in the lymph node (49–52). The SIGLECs were investigated in glioma (53, 54); however, SIGLEC1 was excluded. The previous study showed Sialoadhesin encoded by SIGLEC1 was undetectable in normal human brain microglia, however was intensely detected in perivascular macrophages (55). This enlightened us that parts of the M2 macrophages of glioma were recruited from periphery. Our ICH images acquired from THE HUMAN PROTEIN ATLAS showed the SIGLEC1 was not detected in both normal brain and glioma. A large sample survey is needed to identify the expression of SIGLEC1 in glioma. FPR3 is Formyl peptide receptor 3, which together with other members of Formyl peptide receptor family been implicated in the regulation of tissue repair and angiogenesis (56). In glioma, the Formyl peptide receptor (FPR, also called FPR1) can regulate the invasion, angiogenesis and growth of tumor (57, 58), however, the function of FPR3 in glioma is still unclear. FPR3 was considered to be a pathogen sensor, due to the up-regulated after stimulation with a bacterial endotoxin (59). Interestingly, the migration of CD4+ T cell can be regulated by FPR3 (60). In consideration of FPR3 expression is mainly in monocytes and relates with the grade, IDH status, and prognosis, it is very promising to be a novel biomarker for glioma.

According to the scRNASeq data, we showed some biological progress enriched in specific cells. The phenotype of macrophages was related to whether the cell is neoplastic or not. The M2 macrophages mainly gathered in neoplastic cells, while the M1 macrophages located in non-neoplastic cells. This phenomenon is consistent with previous research (61).

To analysis the scRNASeq data, we found CD163, SIGLEC1, and FPR3 were mainly located in M2 macrophages, the P2RY12 was both detected in M1 and M2 macrophages, nevertheless a large part of P2RY12 were in M1 macrophages. Previous studies have suggested that the cytoplasmic expression of P2RY12 is associated with the expression of M1 markers and low-grade glioma, while the nuclear expression of P2RY12 is associated with the expression of M2 markers and high-grade glioma (33). The level of mRNA expression of P2RY12 may not be used as an indicator to differentiate M1 and M2 macrophages compared with the location of P2RY12 protein in cell. PLAUR and LAPR5 showed inconsistent results between two scRNASeq data. Heterogeneity of glioma makes it difficult to determine the resource of differential expression, both two scRNASeq data only have four couples of samples. For a better understand all the six genes, we analyzed the six genes on the basis of grade, IDH statue, immune score, and stromal score. The expression of LPAR5 had no difference, no matter according to grade nor IDH statue; however, the ability of six genes to predict prognosis in LGG was more efficient than them in GBM. Although we screened the six genes from macrophages, only CD163 in TCGA database can distinguish the high or low of immune score and stromal score. The immune score and stromal score are calculated based on 141 stromal signature genes and 141 immune signature genes respectively, and SIGLEC1 is one of the stromal signature genes. The possible explanation is that too many other stromal and immune that not very important diluted the effect of this six genes. A further research in this field may provide the answer someday. Similarly, the relationship between SIGLEC1 and CD163 in glioma also need to be further studied. In view of the types and proportion of immune cells infiltrated in glioma were different between different grades (62), multi-genes may be a suitable method to evaluate statue of glioma, we constructed a prognostic model by the six genes and verified it. The results showed the prognosis of LGG can be predicted more efficient by this prognostic model than GBM. Especially the ability to predict the outcomes of LGG makes the model a more comprehensive evaluation method, addition with the SIGLEC1 and FPR3 could be two novel biomarkers to estimate grade and IDH status of glioma and six genes are correlated with immune checkpoint, the model will be helping for the diagnosis and treatment of glioma, in particular with respect to evaluate LGG.

## Conclusion

In summary, The six genes construct a prognostic model to predict the outcomes of LGG and are correlated with immune checkpoint which provide a valuable role in diagnosis, prognosis, and immunotherapy of glioma.

## Data Availability Statement

The original contributions presented in the study are included in the article/**Supplementary Materials**. Further inquiries can be directed to the corresponding authors.

## Ethics Statement

The studies involving human participants were reviewed and approved by Renmin Hospital of Wuhan University. The patients/participants provided their written informed consent to participate in this study.

## Author Contributions

YT contributed to publication search, data extraction, and draft writing. YL, TY, and XY contributed to the quality assessment and editing. BL and JY contributed to the statistical analysis. HZ and QC are correspondence authors and contributed equally to the conception and design. All authors contributed to the article and approved the submitted version.

## Funding

The present study was supported by the National Natural Science Foundation of China (No. 81572489).

## Conflict of Interest

The authors declare that the research was conducted in the absence of any commercial or financial relationships that could be construed as a potential conflict of interest.

## Supplementary Material

Supplementary Table 1 A comparison between the previous studies and our study in immune-related genes in glioma.

Supplementary Table 2 The primer sequence of each gene.

Supplementary Figure 1
**(A)** The distribution of top-six biological processes in GSE84465. **(B)** The distribution and expression of six candidate genes in GSE135437.

Supplementary Figure 2
**(A)** The expression of six candidate genes in different immune score group from CGGA. **(B)** The expression of six candidate genes in different immune score group from TCGA. **(C)** The expression of six candidate genes in different stromal score group from CGGA. **(D)** The expression of six candidate genes in different stromal score group from TCGA.

Supplementary Figure 3 Survival analysis of six genes in GBM. **(A)** Kaplan-Meier curves for CD163, LPAR5, PLAUR, FPR3 P2RY12 and SIGLEC1 of GBM in CGGA. **(B)** Kaplan-Meier curves for CD163, LPAR5, PLAUR, FPR3 P2RY12 and SIGLEC1 of GBM in TCGA.

Supplementary Figure 4
**(A)** The ICH images acquired from THE HUMAN PROTEIN ATLAS. **(B)** The number of patients with staining of each protein. **(C)** The mRNA expression of six genes in normal brain and glioma.

Supplementary Figure 5
**(A)** The correlation between six genes and immune checkpoint in TCGA. **(B)** The correlation between six genes and immune checkpoint in CGGA.
