# Supplementary material for: Six Immune Associated Genes Construct Prognostic Model Evaluate Low-Grade Glioma
Source: Front Immunol. 2020 Dec 21;11:606164. doi: 10.3389/fimmu.2020.606164 (PMC7779629; doi:10.3389/fimmu.2020.606164)
Supplement: Supplementary Table 1 — A comparison between the previous studies and our study in immune-related genes in glioma. [file Table_1.docx]

**Table S1 A comparison between the previous studies and our study in immune-related genes in glioma**

| Author | Objects | Data Type | Factors | Databases | Genes |
| --- | --- | --- | --- | --- | --- |
| Xu^[22]^ | LGG | Bulk RNASeq | 1p/19q | CGGA/TCGA | S100A3,FAM19A3,ADM2,CLCF1,  TNFRSF11B,GLP1R,TRDC,F2RL1,  VAV3,BMP8B,NTS,AR,PRLHR |
| Cheng^[23]^ | GBM | Bulk RNASeq |  | CGGA/TCGA | FOXO3,IL6,IL10,ZBTB16,CCL18,  AIMP1,FCGR2B,MMP9 |
| Yin^[15]^ | LGG | Bulk RNASeq | Tumor mutation burden | CGGA/TCGA | BIRC5,CRLF1,GDF15,LTF,PRLHR,  TNFRSF11B |
| Qin^[16]^ | GBM | Bulk RNASeq | TP53 | CGGA/TCGA | S100A8,CXCL1,IGLL5 |
| Yang^[24]^ | GBM | Bulk RNASeq |  | CGGA/TCGA/Rembrandt | TREM1,GBP2,IFITM2,CITA,  TYROBP |
| Zhang^[17]^ | LGG | Bulk RNASeq |  | CGGA/TCGA/GTEx | CANX,HSPA1B,KLRC2,PSMC6,  RFXAP,TAP1 |
| Ni^[18]^ | LGG | Bulk RNASeq |  | TCGA | HRH3,APLNR.FCER1G,SYK,GNG12,GNG13,GNG5,PTPN6,GPR183,  CXCL11,CXCL9,HLA-B,VAMP8,  SSTR1,CCR5,PTAFR,C3,TAS1R1,  ANXA1,OPRK1,ITGB2,CCL5,CXCR3,CXCR4,GNGT2 |
| Zhao^[25]^ | GBM | Bulk RNASeq |  | CGGA/TCGA | CD1D,PTX3,RAC2,ESM1,MDK,  TNFSF14,IL2RB,OSMR |
| Our Study | LGG  GBM  TAMs | Bulk RNASeq  /scRNASeq |  | CGGA/TCGA/GEO | CD163,FPR3,LPAR5,P2RY12,  PLAUR,SIGLEC1 |
