# Supplementary material for: Six Immune Associated Genes Construct Prognostic Model Evaluate Low-Grade Glioma
Source: Front Immunol. 2020 Dec 21;11:606164. doi: 10.3389/fimmu.2020.606164 (PMC7779629; doi:10.3389/fimmu.2020.606164)
Supplement: Supplementary Table 2 — The primer sequence of each gene. [file Table_2.docx]

**Table S2 The primer sequence of each gene**

| Gene | Forward Primer | Reverse Primer |
| --- | --- | --- |
| SIGLEC1 | 5’-CCTCGGGGAGGAACATCCTT-3’ | 5’-AGGCGTACCCCATCCTTGA-3’ |
| CD163 | 5’-GGACATGAGTCCCATCTTTCAC-3' | 5’-AGCTCCACTCTGCCCTCACAC-3’ |
| FPR3 | 5’-CGCACAGTCAACACCATCTG-3’ | 5’-AGCTGTTAAAAAAGGCCAAG-3’ |
| LPAR5 | 5’-GGCCCTGAGGAGGTCTCTG-3’ | 5’-TCATGGCATGGCATTCACCT-3’ |
| P2RY12 | 5’-TCCATTTTGCCCGAATTCC-3’ | 5’-CAGAGTATTTTCAGCAGTGCAGTCA-3’ |
| PLAUR | 5’-GCTTGTGGGAAGAAGGAGAA-3’ | 5’-CCTCGGTAAGGCTGGTGAT-3’ |
| β-actin | 5’- CTGGCACCCAGCACAATG-3’ | 5’- CCGATCCACACGGAGTACTTG-3’ |
