# Supplementary figures and images for: Six Immune Associated Genes Construct Prognostic Model Evaluate Low-Grade Glioma
Source: Front Immunol. 2020 Dec 21;11:606164. doi: 10.3389/fimmu.2020.606164 (PMC7779629; doi:10.3389/fimmu.2020.606164)

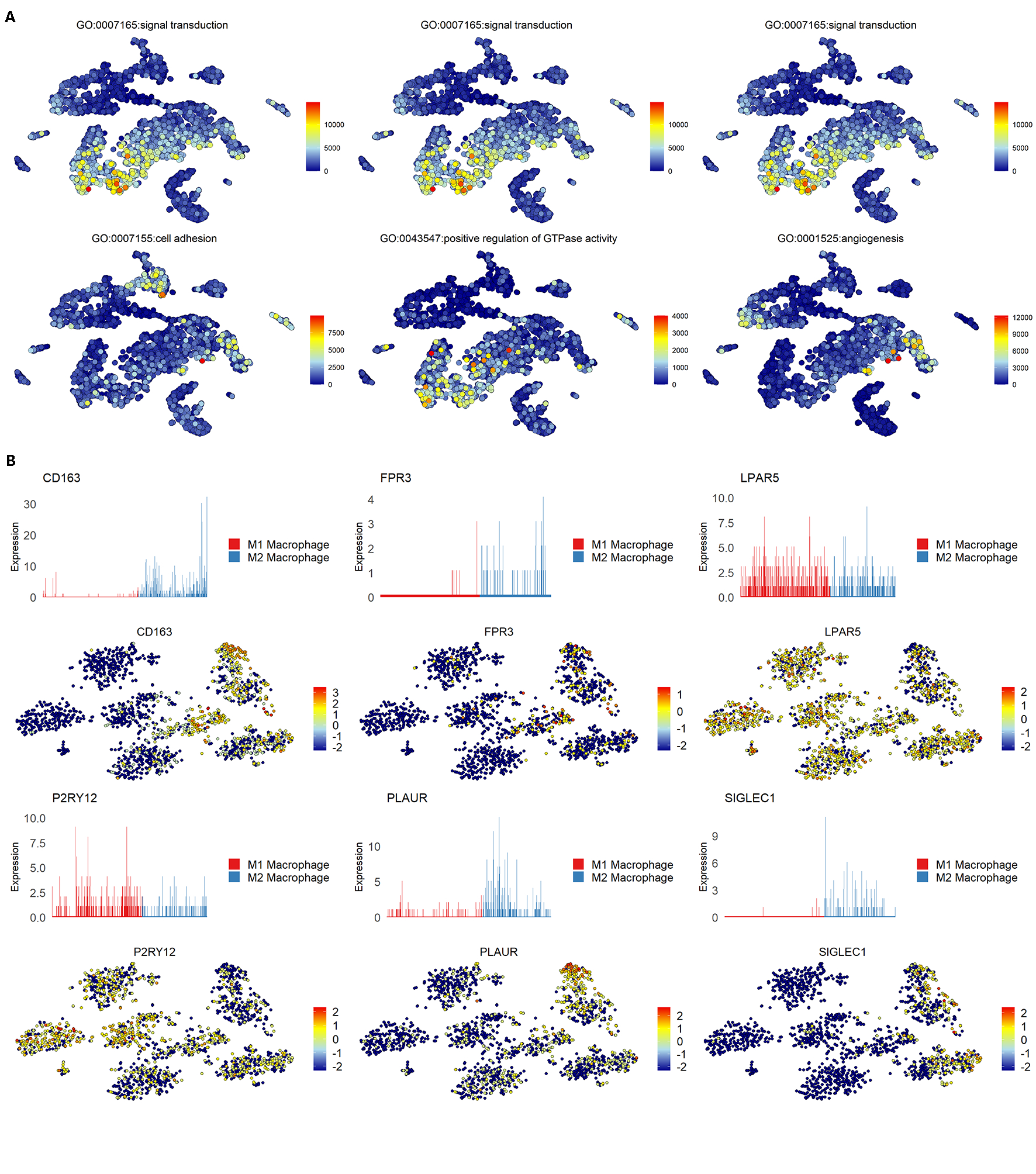

Supplement: Supplementary Figure 1 — (A) The distribution of top-six biological processes in GSE84465. (B) The distribution and expression of six candidate genes in GSE135437. [file Image_1.tiff]

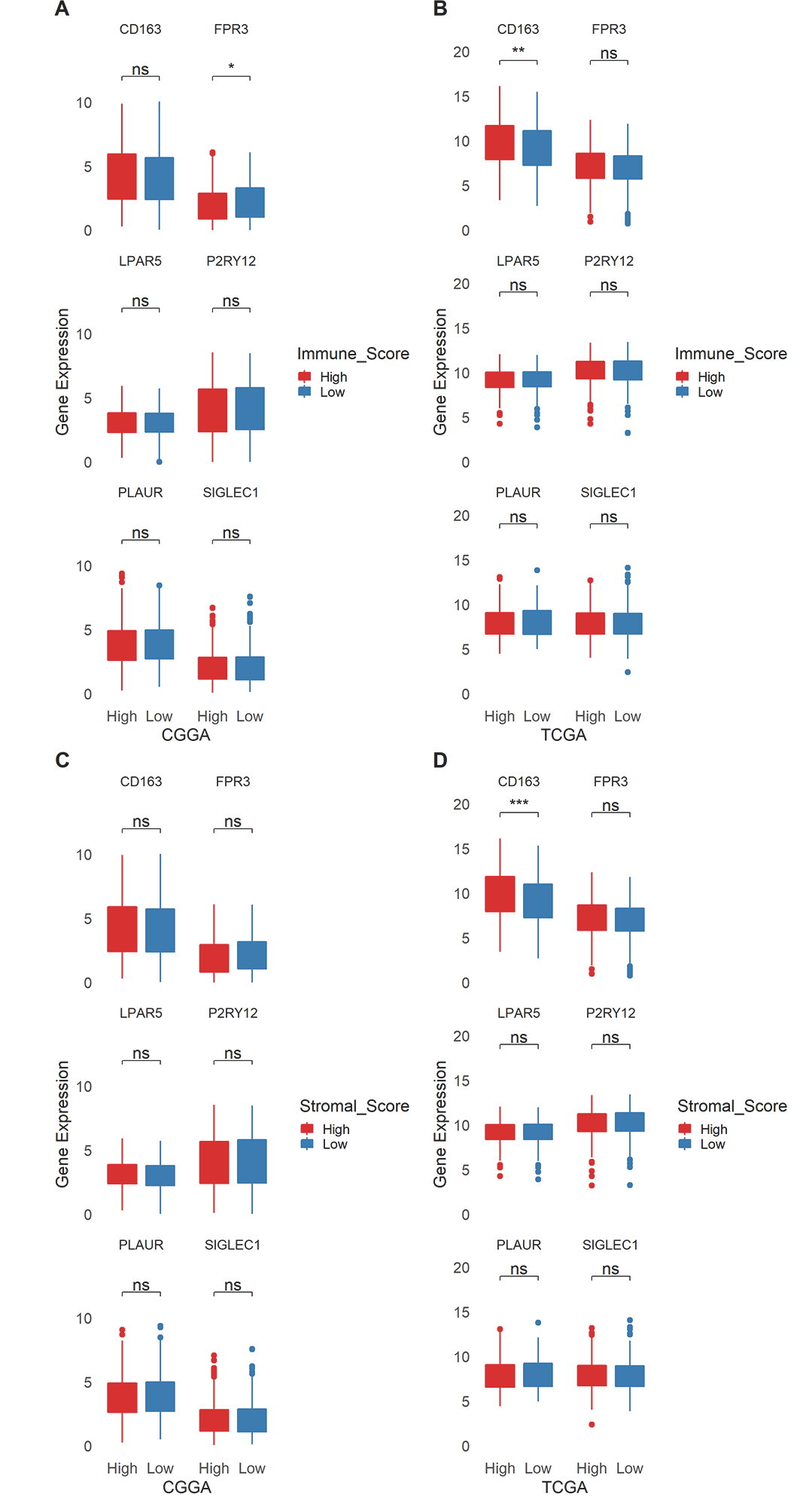

Supplement: Supplementary Figure 2 — (A) The expression of six candidate genes in different immune score group from CGGA. (B) The expression of six candidate genes in different immune score group from TCGA. (C) The expression of six candidate genes in different stromal score group from CGGA. (D) The expression of six candidate genes in different stromal score group from TCGA. [file Image_2.tiff]

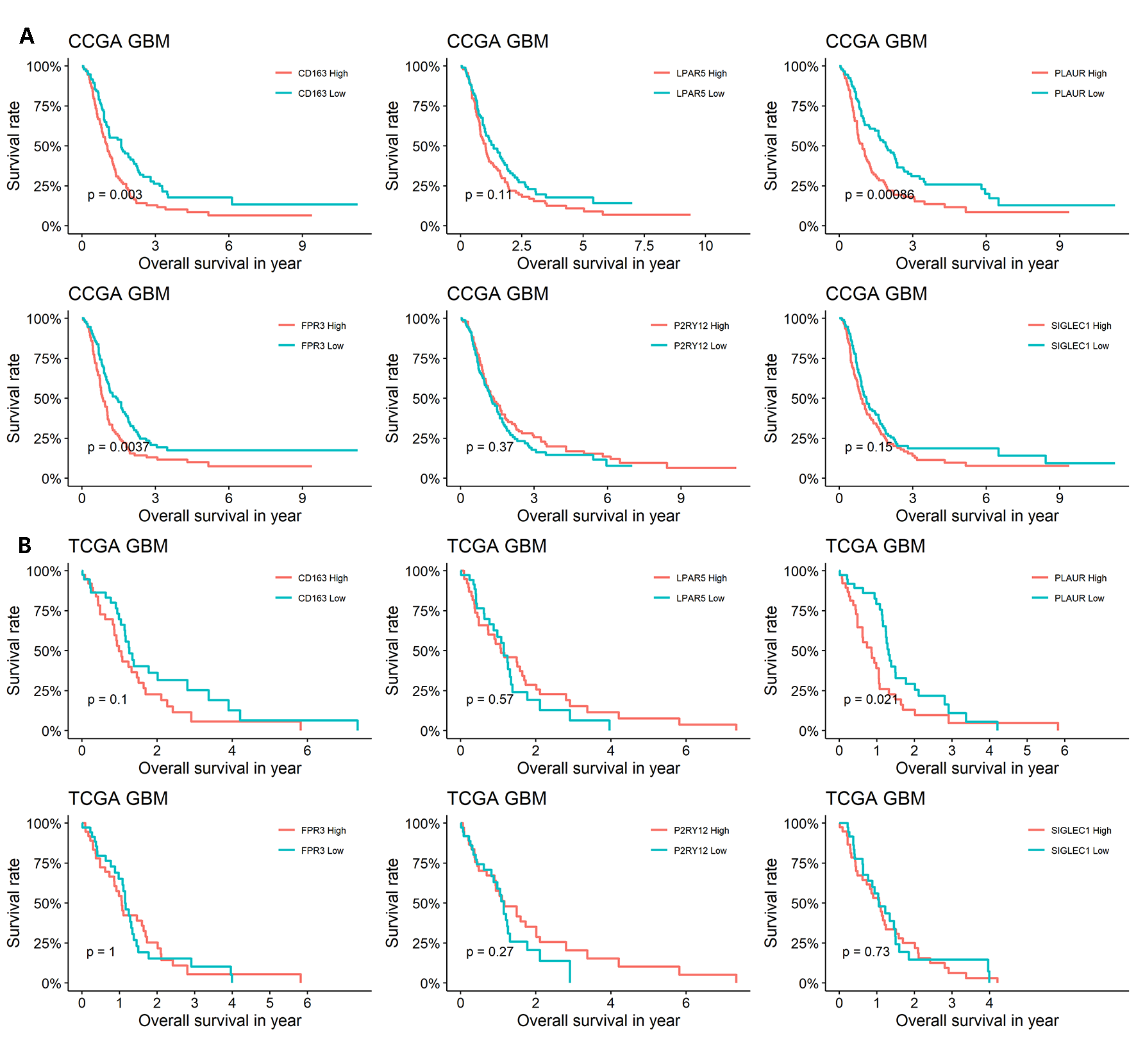

Supplement: Supplementary Figure 3 — Survival analysis of six genes in GBM. (A) Kaplan-Meier curves for CD163, LPAR5, PLAUR, FPR3 P2RY12 and SIGLEC1 of GBM in CGGA. (B) Kaplan-Meier curves for CD163, LPAR5, PLAUR, FPR3 P2RY12 and SIGLEC1 of GBM in TCGA. [file Image_3.tif]

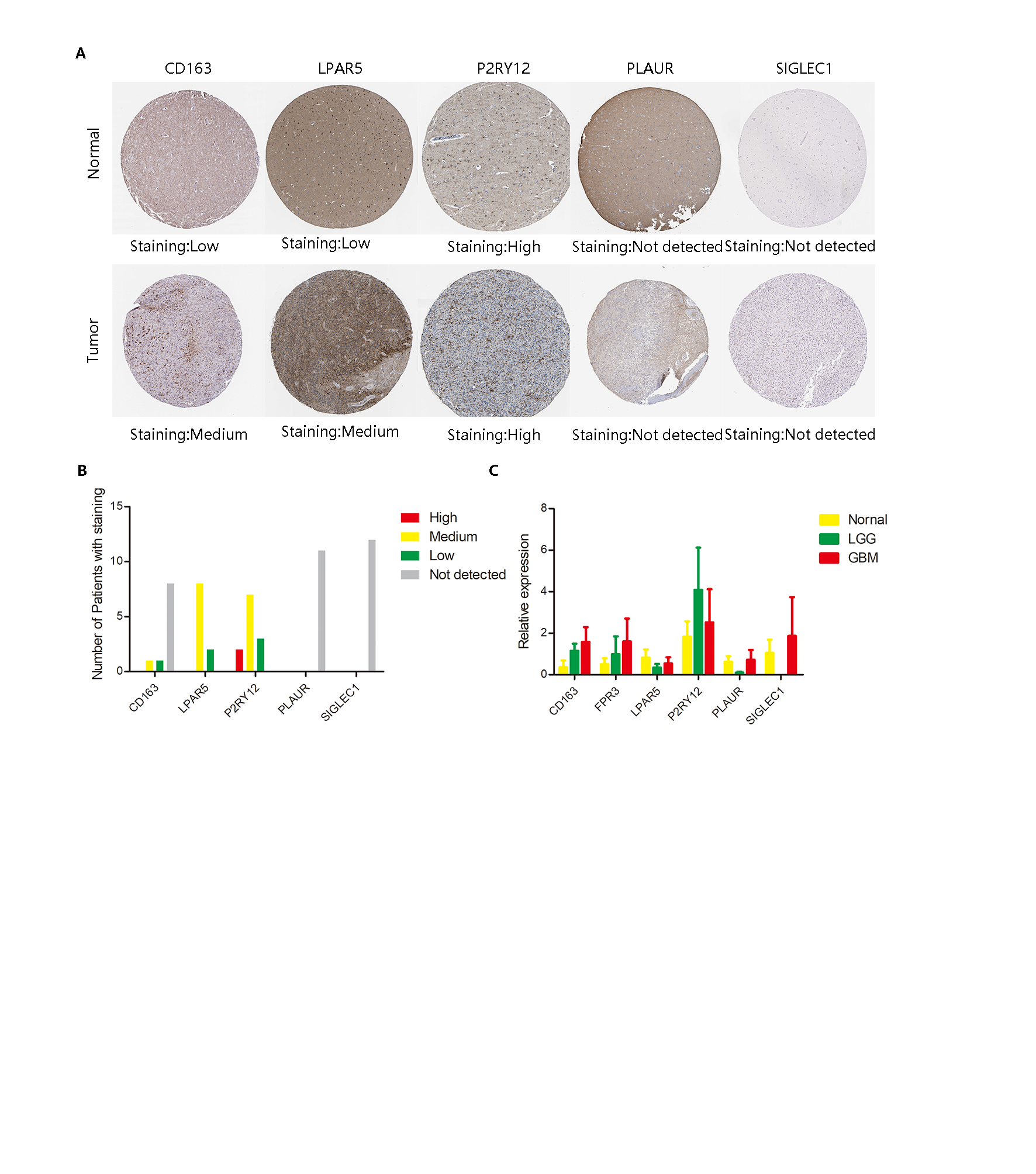

Supplement: Supplementary Figure 4 — (A) The ICH images acquired from THE HUMAN PROTEIN ATLAS. (B) The number of patients with staining of each protein. (C) The mRNA expression of six genes in normal brain and glioma. [file Image_4.tif]

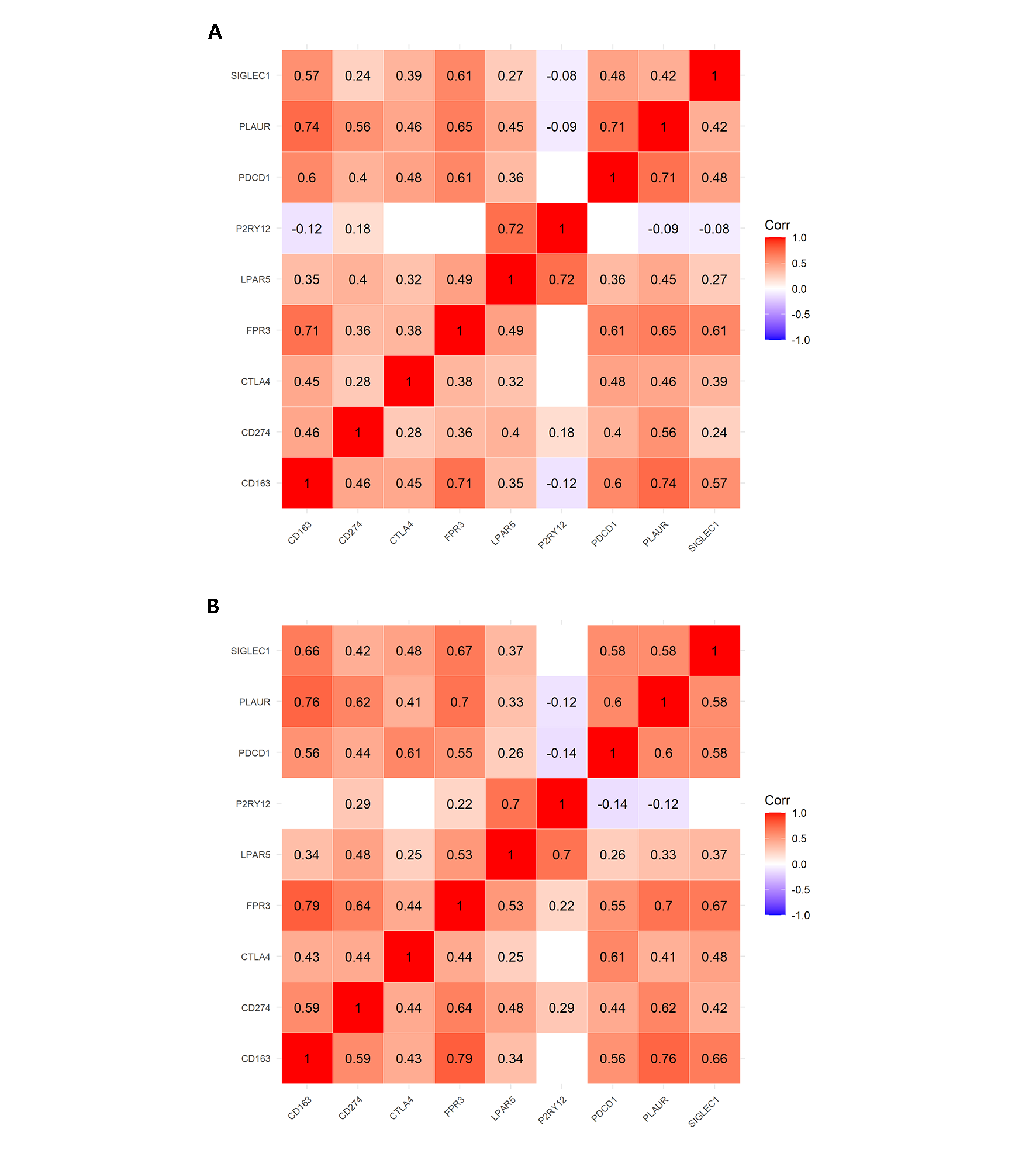

Supplement: Supplementary Figure 5 — (A) The correlation between six genes and immune checkpoint in TCGA. (B) The correlation between six genes and immune checkpoint in CGGA. [file Image_5.tif]
